# MicroRNAs regulate granulosa cells apoptosis and follicular development — A review

**DOI:** 10.5713/ajas.19.0707

**Published:** 2019-12-24

**Authors:** Zhuandi Gong, Juan Yang, Shengju Bai, Suocheng Wei

**Affiliations:** 1 Hospital, Northwest Minzu University, Lanzhou 730030, China; 2 College of Life Science and Engineering, Northwest Minzu University, Lanzhou 730030, China

**Keywords:** microRNAs, Apoptosis, Granulosa Cells, Cumulus Cells, Follicular Development

## Abstract

**Objective:**

MicroRNAs (miRNAs) are the most abundant small RNAs. Approximately 2,000 annotated miRNAs genes have been found to be differentially expressed in ovarian follicles during the follicular development (FD). Many miRNAs exert their regulatory effects on the apoptosis of follicular granulosa cells (FGCs) and FD. However, accurate roles and mechanism of miRNAs regulating apoptosis of FGCs remain undetermined.

**Methods:**

In this review, we summarized the regulatory role of each miRNA or miRNA cluster on FGCs apoptosis and FD on the bases of 41 academic articles retrieved from PubMed and web of science and other databases.

**Results:**

Total of 30 miRNAs and 4 miRNAs clusters in 41 articles were reviewed and summarized in the present article. Twenty nine documents indicated explicitly that 24 miRNAs and miRNAs clusters in 29 articles promoted or induced FGCs apoptosis through their distinctive target genes. The remaining 10 miRNAs and miRNAs of 12 articles inhibited FGCs apoptosis. MiRNAs exerted modulation actions by at least 77 signal pathways during FGCs apoptosis and FD.

**Conclusion:**

We concluded that miRNAs or miRNAs clusters could modulate the apoptosis of GCs (including follicular GCs, mural GCs and cumulus cells) by targeting their specific genes. A great majority of miRNAs show a promoting role on apoptosis of FGCs in mammals. But the accurate mechanism of miRNAs and miRNA clusters has not been well understood. It is necessary to ascertain clearly the role and mechanism of each miRNA or miRNA cluster in the future. Understanding precise functions and mechanisms of miRNAs in FGCs apoptosis and FD will be beneficial in developing new diagnostic and treatment strategies for treating infertility and ovarian diseases in humans and animals.

## INTRODUCTION

The first microRNA (miRNA) was discovered in the *Caenorhabditis elegans* by Ambros and Ruvkun in 1993 [1]. Later, a lot of miRNAs were discovered in human and animals [2]. The miRNAs are originally transcribed from coding genes which occupy 1% to 3% of the genome. Currently, approximately 2,000 annotated miRNAs have been reported in humans [3,4]. The miRNAs regulate about 30% of protein-coding genes in mammals since the different miRNAs may target the same mRNAs [5]. Nucleotides sizes of miRNAs are differentially reported in the mammals, including 19 to 22 nucleotides [6], 20 to 24 nucleotides [6,7], 21 to 23 nucleotide [4], and even 21 to 26 molecules [8]. Consequently, the precise numbers of nucleotides of miRNAs remain undetermined [9,10].

Roughly, 52% of human miRNAs are localized within the intergenic regions, 40% are located within intronic regions, and the rest 8% are situated within exons [11]. In mammals, most miRNAs regulate gene expression via combining the 3′-untranslated region (UTR) and the specific sequences of target mRNAs, causing repression of translation of target mRNAs [12–14]. One miRNA may target hundreds of different mRNAs. However, the regulatory mechanism of every miRNA remains unclearly understood [12].

Follicular granulosa cells (FGCs) play a key role in nourishing oocytes through secreting growth factors and hormones and regulating development of oocytes [15]. It has been well known that miRNAs exert the vital functions in FGCs apoptosis and follicular development (FD) [8,16,17]. The functions of specific miRNAs are implicated in different aspects of FGCs processes of the mammals, such as proliferation [18], differentiation [19], and cumulus expansion [20].

Previous studies aimed to determine the roles of miRNAs on the FD of mammals using various approaches, including conditional knockout of miRNA biogenesis genes, high-throughput sequencing technologies in various animal models. Nowadays, it has been well known that miRNAs exert a significant role in FD and oocyte development of mammals [8,21].

However, so far the accurate effects and regulatory mechanism of different miRNAs regulating apoptosis of granulosa cells (GCs) and FD have still remained unclear, especially their target genes and signaling pathways [20,22]. The present review aimed to comprehensively elaborate the research advances on miRNAs for modulating apoptosis of FGCs and FD in humans and animals so as to seek new diagnostic and treatment scheme for infertility and ovarian diseases.

## miRNAs MODULATE APOPTOSIS OF FOLLICULAR GRANULOSA CELLS

The miRNAs regulate the function of FGCs via altering expression levels of target genes [7,23]. The microRNA (miR)-let-7 family is highly conserved in sequences across animal species. MiR-let-7 family is differentially expressed during follicular atresia [24]. Expression levels of *miR-let-7a*, *let-7b*, *let-7c*, and *let-7i* genes were reduced in early and progressed atretic follicles as compared to those in healthy follicles [25,26]. The miR-let-7g-mediated suppression of mitogen-activated protein kinase kinase kinase 1 (*MAP3K1*) resulted in the expression and dephosphorylation of the transcription factor fork head O1 (*FOXO1*) which induced FGCs apoptosis [27]. Overexpression of miR-let-7g increased the apoptosis rate of the mouse FGCs [26] and *FOXO1* expression in FGCs, and then resulted in nuclear accumulation of dephosphorylated *FOXO1*. Additionally, the expression levels of the apoptosis-associated genes including Caspase 3, BCL2-Associated X (*BAX*), and BES1-interacting Myc-like protein (*BIM*) were significantly upregulated after miR-let-7g mimic was transfected into porcine FGCs. But the anti-apoptotic genes B-cell lymphoma-2 (*Bcl-2*) and myeloid cell leukemia-1 were significantly down-regulated [26]. Briefly, the miR-let-7 family exerted a potential in the regulation of FGCs apoptosis.

MiR-21 is one of three highly luteinizing hormone (LH) -induced miRNAs in murine FGCs [14]. It acts as an antiapoptotic factor in GCs. A loss of miR-21 *in vivo* leads to a reduction of ovulation rates [28]. MiR-21 blocks the apoptosis of murine FGCs [14,29]. Several miR-21 target transcripts have been identified to explain its antiapoptotic effect, including programmed cell death 4, phosphatase and tensin homologue [29,30].

The levels of the primary transcript of miR-21 (pri-miR-21) and mature miR-21 were obviously increased in the cumulus oocyte complexes (COCs) over the maturation period. The pri-miR-21 expression was remarkably decreased in COCs treated with a signal transducer and activator of transcription 3 pathway inhibitor, and cumulus expansion may be prevented. Inihibition of Pri-miR-21 expression directly influenced miR-21 expression in bovine oocytes and cumulus cells (CCs) [31]. Upregulating miR-21 expression significantly reduced CCs apoptosis. The oocyte-secreted factors (OSFs) upregulated miR-21 expression and suppressed FCCs apoptosis by activating the PI3K/Akt signal [29]. It is known that oocytes and CCs are more resistant to apoptosis than other compartments of the antral follicle. However, little is known about the intracellular mechanisms by which OSFs render FCCs resistant to apoptosis [29,32].

MiR-146a is implicated in ovarian cancer development by suppressing the expression of antiapoptotic genes, such as X-linked inhibitor of apoptosis protein, Bcl-2-like protein 2, and baculoviral IAP repeat containing 5 [33]. The downregulation of miR-146a inhibited apoptosis of FGCs by simultaneously targeting interleukin-1 receptor-associated kinase (IRAK1) [34]. A recent study demonstrated that miR-126 inhibited FSH receptor (a direct target gene) expression and increased the apoptosis rate of porcine FGCs [35]. However, the cell apoptosis rate was dramatically reduced when miR-141-3p was overexpressed in rat FGCs [36].

An earlier report revealed that miR-26b enhanced DNA breaks and FGCs apoptosis by targeting the ataxia telangiectasia mutated (*ATM*) gene [37]. Overexpression of miR-26b promoted porcine FGCs apoptosis by regulating the expression of Sma-and Mad-related 4 (SMAD4). These results strongly suggest that miR-26b plays a crucial role in FGCs apoptosis [37]. MiR-125b regulated apoptosis by targeting bone morphogenetic protein receptor 1B (*BMPR1B*) in yak FGCs [38].

The miR-144 was differentially expressed in the porcine preovulatory follicles. The miR-144 regulated FGCs apoptosis and affected follicular atresia [39]. Additionally, miR-224 was involved in the mouse FGCs proliferation via targeting *SMAD4* [33]. Another study indicated that miR-1275 was expressed during the porcine follicular atresia. The miR-1275 can promote early apoptosis of porcine FGCs and the initiation of follicular atresia (FA) by inhibiting estradiol release and expression of liver receptor homolog (LRH)-1 that was bound to the cytochrome P450, family 19, subfamily A, polypeptide 1 promoter and increased its activity. Additionally, miR-1275 attenuated *LRH-1* expression by directly binding to its 3′ UTR [40].

## MiRNAs REGULATE DEVELOPMENT OF MURAL GCs AND CUMULUS CELLS

The GCs are divided and differentiated into mural GCs and CCs that tightly surround the oocyte [41,42]. Both mural GCs and CCs are two specialized cell types that differentiate from a common progenitor during folliculogenesis [43]. Mural FGCs supported the oocytes *via* endocrine and paracrine pathways [44]. The miRNAs are differentially expressed between CCs and mural GCs [45]. Another report showed that 59 miRNAs were found differentially expressed between bovine immature and maturated oocytes [46]. The sequencing analysis revealed the expression of several hundreds of miRNAs in mural GCs and CCs. 53 miRNAs (such as miR-146a-5p, miR-149-5p, miR-509-3p, and miR-182-5p) were differentially expressed between mural FGCs and CCs [43]. Top 10 most abundant miRNAs in mural GCs and CCs were miR-21-5p, let-7a-5p, let-7f-5p, miR-26a-5p, let-7b-5p, let-7g-5p, miR-103a-3p, miR-125a-5p, miR-92a-3p, miR-320a, and other miRNAs. MiR-146a-5p, miR-182-5p, miR-509-3p, and miR-149-5p exert their regulatory functions through 37, 43, 2, and 9 target genes, respectively [43].

Expression of miR-130b was altered during oocyte maturation by directly targeting *SMAD5* and mitogen- and stress-activated protein kinase 1 which were identified as target genes of miR-130b. Overexpression of miR-130b increased the proliferation of mural GCs and CCs. But, inhibition of miR-130b expression during *in vitro* maturation (IVM) of oocytes decreased the first polar body extrusion and the mitochondrial activity. Such, functional modulation of miR-130b affected the proliferation and survival of GC and CC as well as oocyte maturation [47].

Previous studies also demonstrated miR-146a-5p promoting apoptosis of mural GCs by directly targeting *IRAK1* and tumor necrosis factor receptor-associated factor 6 and miR-503-5p inhibiting proliferation by targeting cyclin D2 [48]. In 9 differentially expressed miRNAs, 4 miRNAs (hsa-miR-146a-5p, has-miR-10b-5p, hsa-miR-29b-3p, and hsa-miR-142-5p) in mural GCs, and 5 miRNAs (hsa-let-7c-5p, hsa-miR-125b-5p, hsa-miR-1275, hsa-miR-129-5p, and hsa-miR-129-2-3p) in CC were upregulated [43].

Overall together, studies on cell communication, extracellular matrix and signaling pathways have demonstrated the differential expressions of miRNAs have relevance with physiological functions of CCs and mural GCs [45].

## MiRNAs REGULATE DEVELOPMENT OF OVARIAN FOLLICLES

Both FD and oocyte maturation are completed in the ovaries of female mammals. A highly complicated, spontaneous death phenomenon that is called as atresia takes place during the FD and maturation in the mammals. Follicular atresia is resulted from the apoptosis of GCs surrounding oocytes [49]. In mammals, less than 1% of ovarian follicles will eventually ovulate. More than 99% of ovarian follicles are disappeared as a result of atresia, which affects all stages of follicular growth and development [50].

The FD is mediated by various regulatory factors including many miRNAs [51]. Numerous miRNAs play important roles in follicular atresia and development [49,52]. The miRNAs exert their functions as mediators of these processes via their extensive involvement in post-transcriptional mRNA regulation [53,54]. The miRNAs are differentially expressed during the primordial development [55], luteal development [56] and the whole FD [54].

MiR-378 could affect oocyte IVM by inhibiting the expansion and altering gene expression of CCs, and adjust in estradiol production by depressing aromatase translation in porcine FGCs. The miR-378 decreased IVM rate, suppressed the expression of genes associated with FD, such as bone morphogenetic protein 15 and growth differentiation factor 9 and also increased apoptosis rate [21] since miR-378 targeted to the 3′-UTR of aromatase mRNA [57].

MiR-23a and miR-27a have been reported to promote GC apoptosis by targeting *SMAD5* through the FasL-Fas-mediated pathway [8,58,59]. Knocking down *SMAD5* expression increased the rate of apoptosis [59]. Additionally, studies indicated miR-183-5p and miR-149-5p inhibit the release of progesterone and estradiol, respectively [17]. Moreover, miR-509-3p promotes estradiol secretion by targeting *MAP3K8* [60].

In summary, up to date many miRNAs mediate the process of oocyte maturation and folliculogenesis and also regulate follicular atresia through their target genes, thereby modulating FGCs apoptosis [59]. A large number of miRNAs and miRNA clusters involved in the FD have been documented [61–63]. However, accurate roles of miRNAs and miRNA clusters in this process are not clearly understood [8,63]. Understanding the miRNAs roles will elucidate clearly the mechanisms of GC apoptosis, development and atresia of ovarian follicles [64].

## MiRNA CLUSTERS REGULATE FGCs APOPTOSIS AND FOLLICULAR DEVELOPMENT

A miRNA cluster comprises of more than two miRNAs with similar functions [65]. Currently, it has been reported that the specific miRNA families and clusters are involved in follicular atresia and development including miR-21, miR-23a, miR-145, miR-503, miR-224, miR-383, miR-378, miR-132, miR-212, the let-7 family, miR-17-92 cluster, miR-23-27-24 cluster, miR-183-96-182 cluster, miR-17-92 cluster and so on [28,64,66,67]. However, it has been undetermined which miRNA cluster(s) are associated with the each stage of FD [61,63,64]. Furthermore, actual roles of these miRNA clusters in the FD, atresia and ovulation remain unclear [8,43,68].

Fifteen different miRNAs were found during the growth and selection of dominant follicles [69]. Six miRNAs, including miR-17, miR-18a, miR-19a, miR-20a, miR19b, and miR-92a, are encoded by a single miR-17-92 transcript [70], and are expressed and processed together as a cluster [63]. The miR-17-92 cluster was differentially expressed in GCs from subordinate and dominant follicles at day 19 of the estrous cycle [71]. The overexpression of the miR-17-92 cluster promoted GC proliferation and reduced the proportion of differentiated cells. However, miR-17-92 cluster inhibition resulted in decreased proliferation and increased differentiation in GCs [71].

The miR-183-96-182 cluster (miR-183, miR-96, and miR-182) is highly conserved [72], it is also abundantly expressed in both luteal cells and bovine FGCs of preovulatory dominant follicles [20,73]. This miRNAs cluster impacted bFGCs proliferation. The overexpression of miR-183-96-182 promoted the proliferation of bovine FGCs [20]. This cluster targeted the 3′-UTR of the *FOXO1* gene [74], and thus regulated FD and luteal development via exerting effects on cell survival and steroid production. Moreover, it was also reported miR-182 inhibited FGCs apoptosis by targeting *SMAD7*. However, the actual roles and mechanism of miRNAs remain to be comprehensively investigated in the FGCs apoptosis and follicular atresia [8,75,76].

The miR-23-27-24 cluster comprises the miR-23a gene cluster (miR-23a, miR-27a, and miR-24-2 genes) and the miR-23b cluster (mir-23b, mir-27b, and mir-24-1 genes) that exert their function via *SMAD5*. *SMAD5* is a direct target of mir-23a and mir-27a, which promote GC apoptosis via the Fas-FasL pathway [59]. These evidences suggest that miR-23-27-24 clusters play a role in follicular atresia. On the other hand, expression levels of miR-23a-27a-24, miR-222-221, and miR-214-199a clusters showed an increase until the mid-luteal phase, but expression decreased in the dominant FGCs during the late follicular phase of the estrous cycle.

The miR-17-92 cluster (including miR-17, miR-18a, miR-19a, miR-19b, miR-20a, and miR-92a) was activated *via* direct binding the MYCN proto-oncogene/MYC proto-oncogene promoter [64,77]. This cluster showed to regulate the transforming growth factor β (TGFβ) pathway and affect FGCs apoptosis and follicular atresia [70]. Similarly, the miR-132-212 cluster is associated with ovulation and was elevated after the induction with an ovulatory dose of LH/human chorionic gonadotropin [16], preventing cells from entering into apoptosis.

Based on the reported information in recent years, the regulatory roles of miRNAs on FGCs are summarized in Table 1. As presented in table 1, in total of 41 academic theses regarding 34 miRNAs and miRNAs clusters that reported the regulatory effects of miRNAs on FGCs apoptosis in mammals. The documents indicated explicitly that 24 miRNAs and miRNAs clusters in 29 articles promoted or induced FGCs apoptosis through their distinctive target genes. Seven miRNAs inhibited FGCs apoptosis. So far, the regulatory roles of the remaining 9 miRNAs and miRNAs clusters have been undetermined. We could conclude that a majority of miRNAs show promoting role on apoptosis of FGCs in mammals. But the accurate mechanism of miRNAs and miRNA clusters have been not well understood.

## MOLECULAR SIGNALING PATHWAYS WERE SUMMARIZED

The existence of miRNAs was discovered more than 20 years ago, and since then considerable achievements have been made in understanding the molecular mechanisms in the apoptosis, proliferation and development of follicular cells [78]. MiRNAs can combine with complementary sequences in the 5′-UTR [52] or 3′-UTR [21] of target mRNAs, therefore degrading the mRNA or repressing translation.

**Figure 1:**
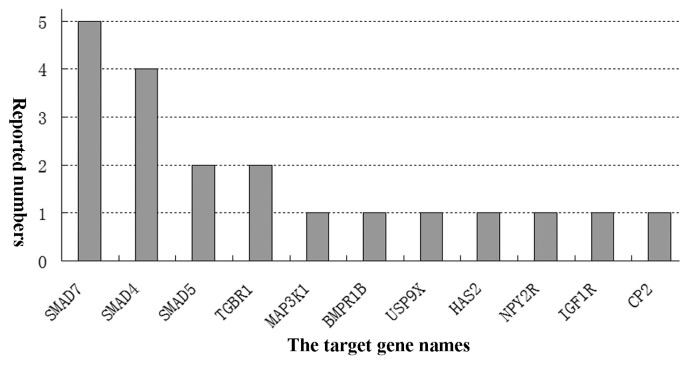
The main target genes and numbers of miRNAs regulatory roles on follicular granulosa cells (FGCs) apoptosis (articles published untill March 2019). The diverse types of miRNAs regulated FGCs apoptosis of and follicular development in humans and animals. But, which target gene is the key gene in these comprehensive processes so far has been undetermined.

Nowadays, it has been known that many miRNAs modulate and FGCs apoptosis and follicular atresia through distinct signaling pathways [61]. Individual miRNAs target multiple genes and involve different patterns of pathways to regulate apoptosis of FGCs, follicular atresia and development. The miRNAs regulate varying signaling pathways of FGCs apoptosis and the ensuing FA via interacting with the mRNAs of target genes [37,58]. Several miRNAs targeting signaling pathways of FGCs apoptosis have been identified in bovine FGCs [20,79]. A previous report showed that at least 77 signaling pathways were reported in the documents which involved miRNAs regulation on FGCs apoptosis and FD [8]. Another study indicated 10 differentially expressed miRNAs and 117 pathways in dominant follicles were collected [51]. An earlier bioinformatic analysis of miRNAs expression of FGCs showed that 139 associated pathways were screened out during the growth and selection of dominant follicles [69]. Furthermore, Cha et al 83 reported that 48 signal transduction pathways are up-regulated by miRNAs and 29 pathways are down-regulated by the miRNAs [80]. Nowdays, it has been clearly undetermined how many signal transduction pathways are involved in the miRNAs regulation roles [8,64]. Based on the documents, partial miRNAs and their signal pathways are summarized in Table 2. As shown in this table, 16 miRNAs exert their functions by targeting 11 genes (mainly *SMAD7*) via mainly 11 signal pathways with the maximum of TGF-β and Bcl-2 (Figure 1).

Moreover, the SMAD played an important role in regulating FD [51,81]. SMAD proteins can transduce the TGF-β family signals at the cell surface into gene regulation in the nucleus. The miR-23a and miR-27a targeted *SMAD5* and regulated apoptosis in human GCs *via* the FasL-Fas pathway [59].

The miR-224 and miR-26b regulate the pathway by targeting *SMAD4* [13,82]. Earlier research indicated that miR-26b was upregulated during porcine follicular atresia. *In vitro* study revealed miR-26b enhanced DNA breaks and GC apoptosis by targeting ATM [83]. Overexpression of miR-26b in follicular FGCs suppressed levels of *SMAD4* mRNA and protein, leading to down-regulation of the antiapoptosis *Bcl-2* gene and the promotion of GC apoptosis [49]. Another study reported that miR-26b and its overexpression could promote apoptosis of porcine FGCs by directly and indirectly targeting *SMAD4*, ubiquitin-specific proteases 9X and hyaluronic acid synthase 2 (*HAS2*) respectively [83]. The apoptosis processes are mediated through the HAS2-CDD44-Caspase-3 pathway [84]. These results strongly suggested that miR-26b plays a crucial role in GC apoptosis and follicular atresia.

The miR-23a and miR-27a promote human GC apoptosis by targeting *SMAD5*. Similarly, miR-92a, miR-181b, and miR-182 directly bind to SMAD7 [47,76], which is considered an antagonist of the TGFβ pathway [17] and an amplifier of TGFβ-induced apoptosis [24]. Roles of the TGFβ pathway and related miRNA regulation have been frequently reported in recent years.

The functional networks play critical roles in the FD which contribute to the profound exploration on miRNAs roles. However, the association with downstream apoptosis genes and proteins remains still unclear [85,86]. The exact signal pathways in which the miRNAs exert need to be investigated in the future [40].

## CONCLUSIONS AND PERSPECTIVES

MiRNAs are involved in physiological and developmental processes by post-transcriptionally inhibiting gene expression. In this review of 41 academic theses, we summarize the current advances in the regulatory roles of miRNAs and miRNA clusters on the FGCs apoptosis and FD in the mammals. Total of 30 miRNAs and 4 miRNAs clusters were reported in all articles. The documents indicated explicitly that 24 miRNAs and miRNAs clusters in 29 articles promoted or induced FGCs apoptosis through their distinctive target genes. The remaining 12 papers reported that 10 miRNAs and miRNAs clusters inhibited FGCs apoptosis. We could conclude that miRNAs or miRNAs clusters could modulate the apoptosis of GCs (including follicular GCs, mural GCs, and cumulus cells) by targeting its specific genes through the different signal pathway. A majority of miRNAs show promoting role on apoptosis of FGCs in mammals. But the accurate mechanism of miRNAs and miRNA clusters is not well understood [8,43,64]. The current results in the published documewnts are still not to clearly eaplain the distinctive effects of each miRNAs or miRNA cluster on FGCs apoptosis and FD in mamnals. It is extremely necessary to ascertain clearly the role and mechanism of each miRNA or miRNA cluster in the future. Understanding comprehensively mechanism of miRNA action may enhance the development of new tools to study miRNAs functions and inspire new diagnostic and treatment strategy or scheme for infertility [87], ovarian disorders and ovarian diseases associated to miRNA high expression or insufficiency [88], such as follicular infertility and ovarian cancer [89].

## Figures and Tables

**Table 1:** Regulatory roles of miRNAs on apoptosis of granulosa cells

Gene symbol	Functions and changes during apoptosis	Target genes	Model species	Study types	References
miR-21	Decreased cleaved caspase 3, inhibited apoptosis, increases ovulation rate	*LNA-21*	Mice	*In vivo*	Carletti [14]
miR-182	Inhibits FGCs apoptosis	*SMAD7*	Human	*In vitro*	Sinha [47]
miR-23a	Increased cleaved caspase-3, decreased caspase-3 protein and promoted FGC apoptosis	*XIAP* (protein)	Human	*In vitro*	Yang [58]
	Promoted FGCs apoptosis in human	*SMAD5*	Human	*In vitro*	Nie [59]
miR-26b	Increases DNA break, inhibits ATM, and promotes FGC apoptosis	*ATM*	Pig	*In vitro*	Liu [37]
	Inhibits Bcl-2, suppresses SMAD4, and promotes GC apoptosis	*SMAD4*	Pig	*In vitro*	Liu [37]
	Suppresses HAS2, enhances caspase-3 and promotes GC apoptosis	*HAS2*	Pig	*In vitro*	Liu [84]
miR-34a	Represses INHBB and promotes GC apoptosis	*INHBB*	Pig	*In vitro*	Tu [90]
miR-92a	Inhibits SMAD7 and promotes apoptosis	*SMAD7*	Pig	*In vitro*	Liu [83]
miR-27a	Increases expression of cleaved caspase-8, cleaved caspase-3, promotes FGC apoptosis	*SMAD5*	Human	*In vitro*	Nie [59]
let-7g	Inhibits MAPK1 and induces GC apoptosis	*MAP3K1*	Pig	*In vitro*	Zhou [25]
	Inhibits TGB-β1 and induces GC apoptosis	*TGBR1*	Pig	*In vitro*	Cao [26]
	Induces FGCs apoptosis	*MAP3K1*	Pig		Cao [32]
	Overexpression of let-7g increase the apoptosis rate of FGCs	*IGF1R*	Mice	*In vitro*	Zhou [23]
miR-125a	Enhances cleaved caspase-3 and promotes FGC apoptosis	*STAT3*	Mice	*In vitro*	Wang [70]
miR-320	Inhibits E2 synthesis and FGC proliferation	*E2f1/Sf-1*	Mouse	*In vitro*	Andrei [43]
miR-15a	promotes release of progesterone and testosterone	*unknown*	Human	*In vitro*	Sirotkin [17]
miR-146a	Inhibits ovarian granulosa cell apoptosis	*IRAK1*	Human	*In vivo*	Chen [34]
miR-146a-5p	Promotes apoptosis of mural gcs	*IRAK1 TRAF6*	Human	*In vivo*	Lei [48]
miR-125a-5p	Promotes GC apoptosis	*Stat3*	Mouse	*In vitro*	Andrei [43]
miR-126^*^	Inhibits FSHR expression and increases the rate of AR-induced apoptosis in FGCs	*FSHR*	Pig	*In vitro*	Du [35]
miR-378	Decreases E2 production	*CYP19A1*	Porcine	*In vitro*	Xu [57]
miR-378-3p	Inhibited FGC differentiation	*PGR*	Bovine	*In vitro*	Sun [21]
miR-224	Enhanced TGF-1-induced FGC proliferation	*TGF-1 Smad4*	Mice	*In vitro*	Shin [13]
miR-10	Suppresses GC proliferation	*BDNF*	Goat	*In vivo*	Andrei [43]
miR-503/322/351 cluster	Reduces of mitochondrial activity in FGCs	*AMAG*	Mouse	*In vitro*	Lei [48]
miR-764-3p	Decreases steroidogenesis	*Sf-1*	Mice	*In vitro*	Wang [70]
miR-22	Suppresses SIRT1 and inhibits FGCs apoptosis	*SIRT1*	Mice	*In vitro*	Xiong [86]
miR-183-96-182 cluster	Promotes GCs apoptosis	*FOXO1*	Cow	*In vitro*	Gebremedhn [73]
miR-17-92 cluster	Promoted GCs proliferation	*PTEN BMPR2*	Cattle	*In vitro*	Andreas [71]
	Affect FGCs apoptosis and follicular atresia	*MYCN/ MYC*	Mice		Wang [70]
MiR-141-3p	Inhibits apoptosis in rat ovarian GCs	*DAPK1*	Rat	*In vitro*	Li [33]
miR-145	Regulates negatively FGC proliferation	*IRS1*	Human	*In vitro*	Naji [28]
	Protects FGCs agaist oxidative stress- induced apoptosis	*KLF4*	Mice	*In vitro*	Zhang [64]
miR-378	Increases apoptosis rate	*Bax/Bcl-2*	Mice	*In vitro*	Sun [21]
miR-16	Suppresses Apoptosis through targeting PDCD4 in polycystic ovarian syndrome	*PDCD4*	Human	*In vivo*	Fu [30]
miR-125b	Regulates apoptosis of FGCs	*BMPR1B*	Yak	*In vivo*	Yao [38]
miR-1275	Promoted early apoptosis of FGCs	*LRH-1*	Pig	*In vitro*	Liu [40]

*LNA-21*, locked nucleic acid; FGCs, follicular granulosa cells; *SMAD4*, *SMAD5*, and *SMAD7*, Sma-and Mad-related 4, 5 and 7, respectively; *XIAP*, X-linked inhibitor of apoptosis protein; *ATM*, ataxia telangiectasia mutated gene; GCs, granulosa cells; *HAS2*, hyaluronic acid synthase 2; *INHBB*, inhibin beta-B; *MAP3K1*, mitogen-activated protein kinase kinase kinase 1; TGB-β1, transforming growth factor-β type 1; *TGBR1*, transforming growth factor-β type 1 receptor; *IGF1R*, insulin-like growth factor 1 receptor; *STAT3*, signal transducer and activator of transcription 3; *IRAK1*, interleukin-1 receptor-associated kinase; *TRAF6*, tumor necrosis factor receptor-associated factor 6; *FSHR*, FSH receptor; *CYP19A1*, cytochrome P450, family 19, subfamily A, polypeptide 1; *PGR*, progesterone receptor; TGF-1, transforming growth factor 1; Smad2, Sma- and Mad-related protein 2; *BDNF*, brain derived neurotrophic factor; *AMAG*, autophagy/mitophagy-associated genes; *Sf-1*, steroidogenic factor-1; *SIRT1*, silent mating-type information regulation 2 homologue 1; *FOXO1*, fork head O1; *PTEN*, phosphatase and tensin homolog deleted on chromosome ten; *BMPR*, bone morphogenetic protein receptor 1b; *MYCN*/*MYC*, Mycn proto-oncogene/Myc proto-oncogene; *DAPK1*, death-associated protein kinase 1; *IRS1*, insulin receptor substrate; *KLF4*, Krüppel-like factor 4; *Bax/Bcl-2*, B-cell lymphoma-2 associated X/B-cell lymphoma-2; *PDCD4*, programmed cell death 4; *BMPR1B*, argeting bone morphogenetic protein receptor 1B; *LRH-1*, liver receptor homolog-1.

**Table 2:** miRNAs and their signal pathways

Signal pathway	mi-RNAs	Target	Model Species	Reference
TGF-β	miR-92a	*SMAD7*	Mouse	Yang [55]
			Human	Donadeu [56]
	miR-181b	*SMAD7*	Pig	Yao [38]
	miR-let-7g	*TGBR1*	Pig	Zhou [25]
	miR-92a	*SMAD7*	Pig	Liu [37]
	miR-182	*SMAD7*	Rat	Luo [15]
	miR-224	*SMAD4*	Mice	Yao [38]
Bcl-2	miR-26b	*SMAD4*	Pig	Worku [49]
	miR-26b	*ATM*	Pig	Liu [84]
	miR-let-7g	*MAP3K1*	Porcine	Cao [32]
	miR-224	*SMAD4*	Pig	Liu [37]
Bcl-2/Bax	miR-125b	*BMPR1B*	Yak	Yao [38]
Bcl-2/MCL-1	miR-let-7g	*TGBR1*	Pig	Cao [32]
FasL-Fas	miR-23a miR-27a	*SMAD5*	Human	Nie [59]
TGFBR1	miR-125b	*SMAD7*	Pig	Yao [38]
HAS2-CDD44-casp-3	miR-26b	*SMAD4*	Pig	Liu [84]
		*USP9X*		Liu [37]
HAS2-HA-cd44-casp-3	miR-26b	*HAS2*	Pig	Liu [84]
sNPFR1/npyr	miR-9a	*NPY2R*	Porcine	Suh [54]
MSK1	miR-130	*SMAD5*	Cattle	Sinha [47]
PKB/mTOR	miR-let-7g	*IGF1R*	Mice	Zhou [23]
CP2/miR-144/COX-2/PGE2	miR-144	*CP2*	Mouse	Zhou [39]

TGF-β, transforming growth factor β; *SMAD4*, *SMAD5*, and *SMAD7*, Sma-and Mad-related 4, 5 and 7, respectively; *TGBR1*, transforming growth factor-beta type 1 receptor; Bcl-2, B-cell lymphoma-2; *ATM*, ataxia telangiectasia mutated; *MAP3K1*, mitogen-activated protein kinase kinase kinase 1; *BMPR1B*, argeting bone morphogenetic protein receptor 1B; MCL-1, myeloid cell leukemia-1 gene; *TGFBR1*, threonine kinase complex composed of type I; HAS2, hyaluronan synthase 2; HA, hyaluronic acid; ubiquitin-specific proteases 9X (USP9X); sNPFR1, short neuropeptide F receptor 1; *NPY2R*, Neuropeptide Y receptor Y2; MSK1, mitogen- and stress-activated protein kinase 1; *IGF1R*, insulin-like growth factor 1 receptor; *CP2*, cyclooxygenase prostaglandin E2; COX, cyclooxygenase; PGE2, prostaglandin.
